# Low-intensity pulsed ultrasound enhances uptake of doxorubicin-loaded gold nanoparticles in cancer cells^☆^

**DOI:** 10.1016/j.ultsonch.2025.107417

**Published:** 2025-06-03

**Authors:** Farshad Moradi Kashkooli, Anshuman Jakhmola, Graham A. Ferrier, Monika Lodyga, Kevin Rod, Jahangir (Jahan) Tavakkoli, Michael C. Kolios

**Affiliations:** a Institute for Biomedical Engineering, Science and Technology (iBEST), a partnership between St. Michael’s Hospital, a site of Unity Health Toronto and Toronto Metropolitan University, Toronto, Ontario, Canada; b Keenan Research Centre for Biomedical Science at St. Michael’s Hospital, Toronto, Ontario, Canada; c Department of Physics, Toronto Metropolitan University, Toronto, Ontario, Canada; d Toronto Poly Clinic Inc., Toronto, Ontario, Canada

**Keywords:** Low-Intensity Pulsed Ultrasound (LIPUS), Gold nanoparticles, Cellular uptake, Enhanced endocytosis, Sonoporation, Hyperthermia, Acoustic radiation force, Targeted drug delivery, Dark field hyperspectral microscopy

## Abstract

The distinctive physicochemical properties of gold nanoparticles (AuNPs), such as biocompatibility, easy functionalization, and a high surface area-to-volume ratio, make AuNPs one of the most suitable candidates for cancer nanomedicine applications. However, achieving efficient uptake of drug-loaded AuNPs into cancer cells has remained a significant challenge in drug delivery. One promising non-invasive, pleiotropic modality that could enhance the cellular uptake of drug-loaded AuNPs by facilitating the transport through cell membranes is low-intensity pulsed ultrasound (LIPUS). This study employs cell experiments (viability and flow cytometry tests), finite element simulations, and dark-field/hyperspectral cell imaging to demonstrate that LIPUS significantly enhances the cellular uptake of doxorubicin-loaded AuNPs and free drug in cancer cells. The synergistic effects of low-intensity ultrasound and therapeutic agents further reduce cell viability, exceeding the effects of ultrasound or doxorubicin-loaded AuNPs alone. Driven by the thermal and mechanical mechanisms induced by LIPUS, this approach enhances endocytosis and sonoporation, thereby increasing cellular uptake of AuNPs and free drug through active and passive transport mechanisms. This results in a substantial improvement in treatment efficacy, marking a promising advancement in targeted drug delivery for cancer therapy.

## 1 Introduction

Precise, targeted, and timely initiation of nano-sized drug delivery systems (NSDDSs) using an external non-invasive stimulus such as low-intensity pulsed ultrasound (LIPUS) can be promising to enhance cancer treatment outcomes beyond conventional chemotherapy or passive NSDDSs alone. Low-intensity pulsed ultrasound can activate drug release from NSDDSs *via* thermal and mechanical effects and overcomes barriers within tumor microenvironments to enable deeper penetration of nanoparticle drug carriers into tumor tissue, thereby reducing adverse effects on normal cells [1,2]. As an unfocused modality, LIPUS targets large regions of interest, typically spanning a few centimeters, at low intensities (spatial-peak time-averaged intensities – I_SPTA_ of a few 100 mW/cm^2^ to a few W/cm^2^) [3].

Thermal effects induced by LIPUS arise from the conversion of acoustic energy to thermal energy, elevating the tumor temperature within the hyperthermia regime (39–45 °C), which can lead to cell membrane disruption, protein denaturation and enhanced permeability of blood microvessels. In contrast, mechanical effects induced by LIPUS encompass phenomena such as radiation pressure, acoustic radiation force (ARF), acoustic streaming, and cavitation [2,4]. Combining both effects can potentially exploit both delivery mechanisms. For instance, our recent research indicates that a combination of thermal and mechanical interactions induced by LIPUS triggers drug release from the surface of AuNPs [5]. Mechanical forces could propel drugs or nanoparticles deeper into cells or tissues. In addition, the mechanical effects of LIPUS induce transient changes in cellular permeability, facilitating enhanced cellular uptake of therapeutic agents [1]. For example, LIPUS can transport plasmonic DNA through the cell membrane, cytoplasm, and ultimately toward the nucleus to facilitate gene therapy [6]. By crossing the transvascular, interstitial, and transmembrane barriers in the tumor microenvironment, LIPUS and nanoparticles have demonstrated efficacy as a relatively emerging and effective standalone treatment [[7], [8], [9]]. Conventional LIPUS operates non-invasively and primarily exerts its biological effects through non-thermal mechanisms. LIPUS can convert mechanical signals into intracellular biological signals by altering cell membrane protein conformation, mechanosensitive ion channels, and conformation of cell membrane receptors. These mechanical stimulations can facilitate the exchange of biomolecules across the cell membrane [9,10]. Both *in vitro* and *in vivo* studies have shown that this exchange can enhance the release of anti-inflammatory cytokines from cells [11,12]. The mechanisms by which ultrasound enhances cellular membrane permeability differ between thermal and mechanical effects. Thermal effects enhance permeability by increasing membrane fluidity [13]. In contrast, mechanical effects caused by ultrasound, such as cavitation, ARF, and microstreaming, provide more transient and reversible means of increasing permeability [8,14,15].

Gold nanoparticles possess notable physicochemical properties, characterized by their small size, high surface area-to-volume ratio, tunable morphology, and excellent biocompatibility and inertness. These all render them highly promising for a wide range of drug delivery applications in oncology [16]. Despite their potential, the efficient delivery of AuNPs to target cancer cells remains a significant challenge, primarily due to physicochemical and biological barriers that impede their cellular uptake, particularly the cell membrane [2,8].

Ultrasound-based therapies, such as focused ultrasound and ultrasound combined with microbubbles, have shown promising results in enhancing drug delivery and therapeutic outcomes [2]. Focused ultrasound allows for precise targeting, but it often risks unintended tissue damage due to its high-intensity energy concentration in a small area [17]. Similarly, microbubble-assisted therapies have demonstrated enhanced drug uptake through acoustic cavitation, but they can introduce operational complexity and increased risks related to the microbubbles themselves, including immune reactions, unintended activation of biochemical pathways or off-target effects [[18], [19], [20]]. In contrast, our study focuses on an unfocused, microbubble-free ultrasound treatment method. Using low-intensity, unfocused ultrasound minimizes the risk of tissue damage and reduces the complexities associated with microbubble use, making the approach simpler, safer, and potentially more adaptable to clinical applications. In our study, we are utilizing spherical AuNPs, which are significantly smaller than microbubbles and have a significantly higher surface area-to-volume ratio [21]. This enables AuNPs to adsorb more drug molecules on their surfaces, while in principle navigating through capillaries and the small interstitial spaces between tissues more readily. This method provides a promising alternative for therapeutic ultrasound, particularly in settings where safety and ease of use are paramount.

The mechanisms underlying ultrasound-enhanced cellular delivery of therapeutic agents are not well-studied. Cellular uptake of ultrasound-mediated nanoparticles primarily occurs through endocytosis [22], the predominant method of cell internalization of macromolecules without external assistance. This process involves the invagination of the plasma membrane, either *via* clathrin-dependent, caveolae-dependent or clathrin/caveolae-independent pathways, resulting in the formation of vesicles that typically fuse with early endosomes [23]. Emerging exogenous stimuli, such as ultrasound, are extensively studied to enhance endocytosis efficiency and improve treatment outcomes [22]. Previous studies have also reported ultrasound-enhanced endocytosis [22,24,25]. Endocytosis is an active process that requires energy in the form of adenosine triphosphate (ATP) to transport substances into the cell by engulfing them with the cell membrane [26]. In contrast, sonoporation is generally considered a passive process as it is triggered by ultrasonic waves (usually interacting with microbubbles), which temporarily create pores in the cell membrane, allowing substances to enter the cell without the cell expending energy directly. Traditional bubble-based sonoporation methods often necessitate specialized contrast agents, adding complexity and cost to the process. Additionally, these methods risk causing irreversible damage to critical cell components, reducing cell viability and compromising the effectiveness of delivered materials [27]. To address these challenges, non-bubble-based sonoporation mechanisms are being developed, including those utilizing ARF and acoustic streaming-induced shear stress [27,28], which will be employed in the current study. In addition to enhanced endocytosis, ultrasound-induced mechanical forces can modulate the physicochemical properties of the cell membrane. This modulation can selectively disrupt chemical bonds in certain proteins or ion channels (*e.g.*, mechanosensitive channels such as Piezo1 and TRPV2 [29]), thereby quantitatively achieving specific biological effects [22].

For successful cancer therapy, therapeutic agents must reach the cancer cells efficiently and in sufficient quantities to eliminate them. Therefore, it is crucial to enhance both the distribution of drugs within the tumor tissue and the cellular uptake of these cancer drugs. This study presents a pioneering application of LIPUS in nanoparticle-based drug delivery, specifically targeting enhanced cellular uptake in cancer therapy. By elucidating the complex interplay between unfocused ultrasound waves, AuNPs, and cancer cells, we aim to gain deeper insights into how ultrasound can overcome cellular barriers and facilitate the intracellular delivery of AuNPs. Using LIPUS to enhance AuNP uptake in MDA-MB-231 breast cancer cells, we uncover key physical mechanisms underpinning this cellular uptake process due to ultrasound-induced hyperthermia, ARF, and acoustic streaming. Our findings demonstrate that LIPUS significantly improves cellular uptake, not only for drug-loaded nanoparticles but also for standalone drug formulations, through enhanced endocytosis and sonoporation. These results are supported by quantitative analyses, including theoretical modeling, cell viability assays, and flow cytometry, alongside qualitative observations using dark-field microscopy, collectively providing compelling evidence of the technique's efficacy. The findings of this study advance the understanding of ultrasound-mediated nanodrug delivery, optimize therapeutic strategies, and establish a transformative framework for the development of highly efficient, targeted AuNP-based cancer treatments.

## 2 Material and methods

### 2.1 Materials

Gold (III) chloride trihydrate (99.9 %), trisodium citrate dihydrate, and 3-(4,5-dimethylthiazol-2-yl)-2,5-diphenyltetrazolium bromide (MTT), as well as 2-(4-amidinophenyl)-6-indolecarbamidine dihydrochloride (DAPI), were sourced from Sigma Aldrich (St. Louis, MO, USA). Doxorubicin (DOX) hydrochloride salt (>99 %) was obtained from LC Laboratories (Woburn, MA, USA). The Annexin V conjugated with Fluorescein Isothiocyanate (Annexin V-FITC) Apoptosis Detection Kit was acquired from BioVision Canada. Roswell Park Memorial Institute (RPMI) 1640 medium, 0.05 % trypsin/0.53 mM Ethylenediaminetetraacetic acid (EDTA), and phosphate-buffered saline 1X (PBS) were purchased from WISENT Inc. (Saint-Jean-Baptiste, QC, Canada). MilliQ® water, produced by a Millipore ultraviolet (UV) ultrapure water purification system (Sigma Aldrich, St. Louis, MO), was used for sample preparation and all experiments. A high-frequency acoustic absorber, Aptflex F28, to mitigate unwanted ultrasonic reflections and provide acoustic isolation, was purchased from Precision Acoustics Inc. (Dorchester, UK).

### 2.2 Gold nanoparticles synthesis and characterization

Gold nanoparticles were synthesized using a method previously patented and developed in our lab [21,30]. Stock aqueous solutions of gold(III) chloride trihydrate (2 mM), trisodium citrate (38.8 mM), and doxorubicin (DOX, 10 mM) were prepared in Milli-Q water. Doxorubicin (DOX), an anthracycline antibiotic, is a widely used broad-spectrum chemotherapeutic agent. To initiate the synthesis, 20 µL of the DOX solution was mixed with 500 µL of the trisodium citrate solution. Next, 500 µL of the gold(III) chloride trihydrate solution was added, and the mixture was rapidly vortexed and left undisturbed at room temperature for a few hours. The initial orange-colored reaction mixture turned brown upon adding gold(III) chloride, and within a few hours, a bright red colloidal solution formed, demonstrating the formation of spherical AuNPs. High-Resolution Transmission Electron Microscopy (HRTEM) and Scanning Transmission Electron Microscopy (STEM) micrographs confirmed their spherical shape, size, and polycrystalline nature. It should be noted that DOX is attached to the surfaces of AuNPs *via* noncovalent bonds as discussed in detail in our previous publications [5,21,31].

**Fig. 1:**
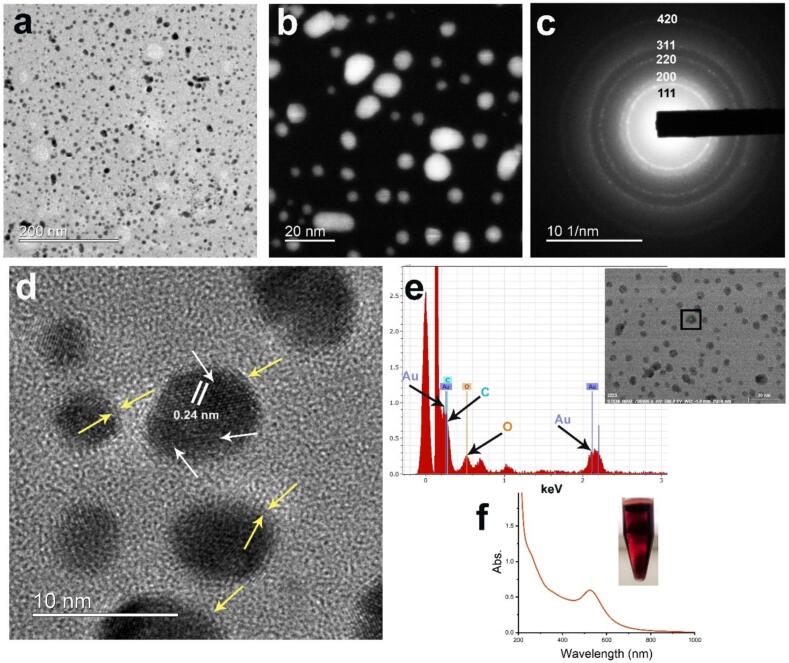
Gold nanoparticle synthesis and characterization. (a) TEM micrograph of AuDOX nanoparticles, (b) STEM micrograph of AuDOX nanoparticles, (c) SAED pattern of AuDOX nanoparticles displaying polycrystalline ringed pattern and *fcc* crystal structure, (d) HRTEM micrograph of AuDOX nanoparticles displaying lattice faults and twin boundaries, white arrows delineate boundaries between different crystalline domains in a single nanoparticle while yellow arrows display the layer of citrate and DOX on the surface of nanoparticles, (e) EDX analysis of a single point on a nanoparticle surface displaying peaks of Au, carbon and oxygen, (f) UV–vis spectrum of colloidal solution of AuDOX nanoparticles and the color of colloidal solution. (For interpretation of the references to color in this figure legend, the reader is referred to the web version of this article.)

The colloidal solution of DOX-loaded AuNPs (AuDOX) was bright red in color, which is characteristic of spherical-shaped nanoparticles, and exhibited a sharp localized surface plasmon resonance (LSPR) band at approximately 530 nm (Fig. 1f). TEM/STEM analysis confirmed the morphology, revealing numerous spherical-shaped nanoparticles with average size of 7–9 nm (Fig. 1a & 1b). Using aberration-corrected HRTEM, the contrast of light elements in nanoparticles can be enhanced [32]. This technique also allows for the visualization of ligand layers, such as citrate and DOX, on the surfaces of the nanoparticles, as indicated by yellow arrows in the micrograph. A closer examination of HRTEM (Fig. 1d) micrographs also revealed lattice defects, stacking faults and dislocations within nanoparticles. The selected area electron diffraction (SAED) patterns (Fig. 1c) demonstrated a polycrystalline structure with a face-centered cubic (*fcc*) crystal lattice. Energy-dispersive X-ray (EDX) analysis at a single point on the nanoparticle surface displayed carbon and oxygen peaks, indicative of surface molecules (citrate and DOX) as depicted in Fig. 1e.

### 2.3 Experimental setup

**Fig. 2:**
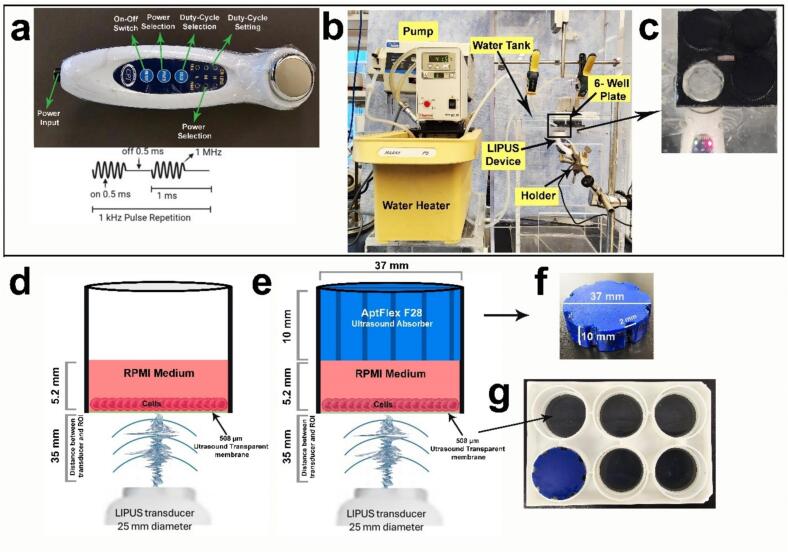
Experimental setup and schematic representation of the low-intensity pulsed ultrasound (LIPUS) device and experimental chamber. (a) The LIPUS transducer with key control features highlighted, including power input, on–off switch, power selection, duty cycle selection, and settings. The waveform represents a 1 MHz signal with a pulse repetition frequency of 1 kHz and a duty cycle of 50 % (0.5 ms on, 0.5 ms off); (b) Complete experimental setup, including a water tank and water heater for temperature regulation, a pump for circulation, a LIPUS device, and a custom holder to position the 6-well plate; (c) Placement of the 6-well plate in the LIPUS device chamber during the experiment; (d) Schematic of the LIPUS target well without an ultrasound absorber, demonstrating a well containing RPMI medium and cells over a 508 µm ultrasound-transparent membrane; (e) Schematic of the LIPUS target well with an AptFlex F28 ultrasound absorber positioned above the RPMI medium to minimize reflections; (f) The fabricated AptFlex F28 absorber disc with dimensions of 37 mm diameter, 2 mm thickness, and a central 10 mm cutout for experimental optimization; (g) A 6-well plate with ultrasound-transparent membranes installed, prepared for exposure to LIPUS with or without the absorber.

The activation of DOX from AuDOX nanoparticles was carried out using an unfocused LIPUS transducer (Fig. 2a) that was clamped, oriented upward, and pressed against a water-sealed mylar window on the floor of a water-filled acrylic tank (Fig. 2b & 2c). An ultrasound-transparent gel devoid of air bubbles and a mylar window enabled the uninterrupted transmission of ultrasound waves upward through the tank floor and water toward a cell monolayer on a well membrane inside a 6-well plate. Within a Bioflex 6-well cell culture plate (Flexcell International, Burlington, NC, USA), different study groups (see Section 2.4) were prepared in separate wells, each containing a cell culture medium and a cell monolayer grown on an ultrasound-transparent rubber membrane (Fig. 2d).

A blue polyurethane high-frequency ultrasound absorber [33], 10 mm in thickness (AptFlex F28), was used to absorb undesired ultrasonic reflections and provide acoustic isolation from the top of the cell culture plate (Fig. 2e). The ultrasound absorber (37 mm in diameter) was precisely designed and shaped using a Glowforge Pro 3D Laser Cutter / Engraver (45 W laser power). Eight peripheral semi-cylindrical grooves (2 mm in diameter) were laser etched on the edges of the absorber. These grooves allowed air to escape when the absorber was fitted into the well upto the meniscus of the cell culture medium (Fig. 2f & g). The temperature at the central region of the cell culture medium was precisely measured using a wire thermocouple inserted through a small hole made at the center of both the plastic cover plate and the absorber. A second wire thermocouple tip was fed through one of the eight peripheral cylindrical grooves to measure the peripheral temperature.

Our patented 1 MHz LIPUS device [34] operated at a 1 kHz pulse-repetition frequency and a 0.5 ms pulse duration, thereby generating 3.2 W of acoustic power. Along the well membrane, this power generates a maximum acoustic pressure of 0.49 MPa and a maximum time-averaged intensity of around 1.7 W/cm^2^, as determined by acoustic simulations in COMSOL Multiphysics®. For each experiment, the LIPUS transducer was run for two consecutive 5-minute intervals for a total of 10 minutes. A peristaltic pump and water heater circulated heated water into the tank, maintaining a local water temperature of 37 °C in the 6-well plate.

### 2.4 Cell culture method and cell viability assays

In this study, a monolayer of MDA-MB-231 breast cancer cells were cultured in a 6-well plate with an ultrasound-transparent, flexible rubber membrane. The membrane which was few microns in thickness enabled maximal transmission of ultrasound energy through the cell layer. All six wells were filled with cell culture medium to a 5.2 mm height (Fig. 2c), and the plate was placed in an incubator at 37 °C and 5 % CO_2_. Phosphate buffered saline (PBS) served as the washing agent for the cells at various stages, while trypsin facilitated the detachment of cells from the seeding membrane.

MDA-MB-231 cancer cells were incubated for two days prior to being subjected to one of seven experimental conditions: 1) control; 2) ultrasound only; 3) DOX only; 4) DOX + ultrasound; 5) AuDOX only; 6) AuDOX + ultrasound; and 7) AuPVA + ultrasound. The seventh condition, involved the replacement of DOX with non-toxic polyvinyl alcohol (PVA) on the gold nanoparticle surface. This was included to determine whether the presence of AuNPs alone had any effect on cell viability in presence of ultrasound. Each experimental condition was repeated atleast three times. Once the cells reached 50% confluency, they were treated with either DOX or AuDOX at a concentration of 9.5 µM, followed by 10 minutes of ultrasound exposure. After a two-day incubation, the cells were washed to remove any residual drug, and MTT and flow cytometry assays for quantifying cell viability were performed.

#### 2.4.1 MTT cell viability assay

The MTT assay measures cellular metabolic activity by assessing the reduction of MTT to purple formazan crystals predominantly by mitochondrial dehydrogenases, providing insights into overall metabolic levels in cells. In this method, MTT is dissolved in RPMI (concentration = 0.84 mg/mL), added to a cell culture, and incubated for 3 h. Then, 4 mL of dimethyl sulfoxide (DMSO) per well was used to dissolve the purple formazan crystals formed by metabolically active cells, enabling spectrophotometric absorbance measurement. This process allows for accurate cell viability quantification based on the colorimetric signal's intensity. The results of the MTT assay are expressed as the percentage of viable cells relative to untreated cells.

#### 2.4.2 Flow cytometry assay

Flow cytometry was used to evaluate the viability of cancer cells following different treatments. Live-dead analysis was conducted using excitation provided by Annexin V-FITC and DAPI filters. Annexin V-FITC is a widely used probe for detecting apoptosis, a form of programmed cell death. Annexin V is a Ca^2+^-dependent phospholipid-binding protein with a high affinity for phosphatidylserine, a membrane phospholipid that relocates and translocates to the outer surface of the cell membrane during apoptosis or cell death [38]. Following our treatment protocol, monolayer cells treated under various experimental conditions were stained, dissociated into single-cell suspensions, and analyzed *via* flow cytometry. The detailed procedure included apoptosis induction with appropriate controls, cell harvesting and suspension, centrifugation, and washing with PBS. Cells were resuspended in 1X Annexin V binding buffer, and 2.5 µL of Annexin V-FITC was added to each 500 µL of cell suspension [39]. After a 15-minute incubation at room temperature, a viability dye (DAPI) was added. Cells were identified as early apoptosis (Annexin V + DAPI −), late apoptosis (Annexin V + DAPI +), necrosis (Annexin V− DAPI +) and live cells (Annexin V− DAPI−) using CytoFLEX-LX (Beckman Coulter, Life Sciences). Data was processed and analyzed using FlowJo software (FlowJo LLC).

### 2.5 CytoViva® imaging to visualize cellular internalization of gold nanoparticles

The CytoViva® hyperspectral microscope employed in this study is tailored for the optical observation and spectral confirmation of nanoparticles as they interact with cells and tissues. The microscope system employs oblique angle illumination, which generates high signal-to-noise ratio dark-field images [[35], [36], [37]]. MDA-MB-231 cell lines were incubated with AuDOX nanoparticles for 24 h after ultrasound exposure to visualize the internalization of nanoparticles within cells. After incubation for 24 h, the cells were washed twice with PBS to eliminate any free nanoparticles. Internalized nanoparticles were visualized using Hyperspectral-Enhanced Dark Field Microscopy (HEDFM) for dark-field imaging.

### 2.6 Ultrasound simulation

**Fig. 3:**
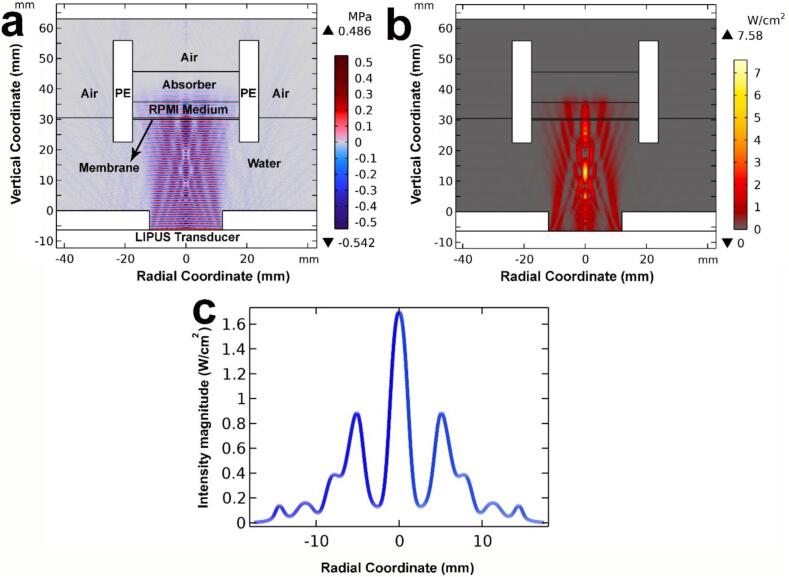
At 3.2 W acoustic power, within a single well, the 2D simulated (a) acoustic pressure distribution and (b) acoustic intensity distribution accumulated near the well membrane where cancer cells were seeded (vertical coordinate = 30 mm); the 1D simulated (c) intensity magnitude distributions along the membrane surface.

In the current study, simulations are crucial in understanding and predicting the system's behavior. The simulations provide detailed insights into the ultrasound field, particularly on the membrane surface where cells are located, which are not easily obtainable through experimental methods alone. Acoustic pressure (Fig. 3a) and intensity (Fig. 3b) distributions within our experimental setup were simulated using the finite element analysis software COMSOL Multiphysics® (Version 6.2). The simulation employed a 2D axisymmetric geometry (Fig. 3a), incorporating various materials (polyethylene oxide, mylar, water, acrylic, and air). The geometry was meshed using a fine triangular mesh, with a maximum element size of 10 μm inside membranes and λ/12 outside membranes. The simulation utilized COMSOL's pressure acoustics module to solve the Helmholtz equation in the frequency domain (Eq. (1) [3,31]), with a normal inward displacement applied to the boundary of the LIPUS transducer:

(1) $$\nabla ∙\left(\frac{1}{\rho }\nabla p\right)+\frac{\kappa^{2}}{\rho }p=0$$

where *p*, *ρ*, *ω* and *κ* are the acoustic pressure, medium's density, angular frequency, and wave number, respectively. For further details on the governing equations for acoustic pressure, intensity, ARF, and mechanical index, please refer to [3,31]. To accurately simulate the acoustic wave reflections, the acrylic tank and polyethylene oxide domains were modeled using COMSOL's solid mechanics module, with their shared boundaries with the remaining domains designated as 'acoustic-structure boundaries'.

Using the acoustic and structural properties of the various materials (Table 1), we conducted simulations involving a single-well setup surrounded by water. An ultrasound absorber (Aptflex F28, Precision Acoustics, UK) placed above the medium prevents ultrasound reflections that would otherwise occur from an air boundary, which would otherwise induce standing waves [31].

**Table 1:** Main material and LIPUS parameters used in the COMSOL simulation. Material parameters were taken from [31,40].

Domain	Parameter	Value	Unit
LIPUS Transducer	Frequency	1.0	MHz
	Acoustic Power	4.12	W
	Displacement	17.48	nm
	Transducer Diameter	24	mm

Water/PBS	Attenuation Coefficient	0.0253	Np/m
	Density	994.23	kg/m^3^
	Speed of Sound	1520.6	m/s

Mylar	Attenuation Coefficient	40	Np/m
	Density	1180	kg/m^3^
	Speed of Sound	2540	m/s

Air	Attenuation Coefficient	18.9	Np/m
	Density	1.184	kg/m^3^
	Speed of Sound	353	m/s

Acrylic	Attenuation Coefficient	14.74	Np/m
	Density	1190	kg/m^3^
	Speed of Sound	2750	m/s
	Young's Modulus	3.2	GPa
	Poisson's Ratio	0.35	−

Polyethylene Oxide	Attenuation Coefficient	18.9	Np/m
	Density	1210	kg/m^3^
	Speed of Sound	2250	m/s
	Young's Modulus	1.0	GPa
	Poisson's Ratio	0.3	−

AptFlex F28 Absorber	Attenuation Coefficient	30	30 dB/cm/MHz
	Density	1010	kg/m^3^
	Speed of Sound	1500	m/s

## 3 Results

A cross-section of pressure and intensity fields along with the intensity distribution across the membrane, where the cells are located (Fig. 3a-c), reveals cross-sectional profiles resembling the free-field minus a small portion resulting from acoustic reflections from the membrane. The majority of ultrasound energy passes through the membrane and becomes absorbed by the ultrasound absorber. Without the absorber, the air boundary can yield an acoustic standing wave with the LIPUS transducer, where the resulting amplitudes highly depend on the distance the acoustic wave travels relative to its wavelength.

**Fig. 4:**
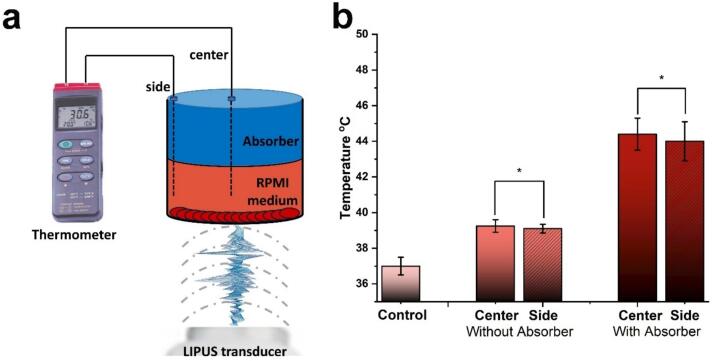
Experimental temperature increases after 10 min of LIPUS exposure. (a) Schematic representation of the temperature measurement setup; (b) Temperature measurements recorded after 10 minutes of LIPUS exposure under two conditions: with and without an absorber in the center and side of the target well. The error bar illustrates the mean ± standard deviation of three experimental measurements. Statistical significance (p-value − non-significant*).

In addition to acoustic pressure and intensity, the presence or absence of the absorber also impacts the temperature of the cell culture medium (RPMI) after 10 minutes of ultrasound exposure (Fig. 4). Due to heating within and from the absorber, the RPMI temperature reached 44.4 °C, the higher end of the mild hyperthermia range. Without the absorber, where the primary acoustic interactions are mechanical, the temperature reached 39.2 °C. Since the wavelength (λ) of the ultrasound is larger than the thickness of the monolayer cells (mm *vs*. µm), and given that the absorption coefficient of the cells is low, the cells primarily experience mechanical effects rather than thermal effects from the ultrasound field. Conversely, the absorber, with its high attenuation coefficient (30 dB/cm/MHz) and the small volume of our target well, effectively converts mechanical energy into thermal energy within the well, as illustrated in Fig. 4b. Furthermore, in our previously published paper [41], an *ex vivo* setup showed that tissue temperature increased to approximately 42 °C with a power setting of 3.29 W.

Depending on the exposure time, normal cells can tolerate temperatures up to 42–45 °C, whereas cancer cells are generally more susceptible to hyperthermia due to dysregulated stress responses. The thermal dose considers both temperature and exposure duration. The threshold for cell death varies depending on tissue type and exposure parameters. A commonly used metric is the cumulative equivalent minutes at 43 °C (CEM43), which integrates temperature and exposure time to assess thermal damage. In our study, the calculated thermal dose remained below the threshold for cell death, indicating that irreversible thermal damage was avoided. Prior research has demonstrated that mild hyperthermia can enhance cell membrane permeability, thereby facilitating processes such as increased drug uptake [42]. In our experiments, we carefully controlled the thermal exposure: the maximum temperatures recorded were 44.4 °C in the presence of an absorber and 39.2 °C in its absence. These exposures were deliberately maintained within a range to minimize cytotoxic effects attributable to heat alone. The corresponding thermal doses, averaged over three independent trials, were approximately 21 min CEM43 in the presence of the absorber and 0.3 min CEM43 in its absence, lower than values known to cause cell death (60–240 CEM43°C) [43,44].

**Fig. 5:**
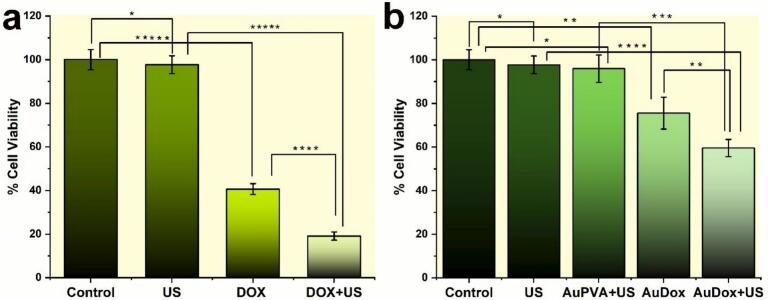
Cell viability test after a 10 min ultrasound exposure. In the presence of an absorber, LIPUS exposure was combined with (a) DOX alone or (b) AuDOX nanoparticles. In the control group, the cell monolayer is neither exposed to ultrasound nor drug. The concentration of DOX is 9.5 µM in all relevant cases. The AuPVA + US group served as a control,representing AuNPs exposed to ultrasound in the absence of drug loading. Error bars represent the standard deviation of three experiments. Statistical significance (p-value- non-significant*, p < 0.01**, p < 0.003***, p < 0.0003****, p < 0.0001*****).

The combination of mechanical and thermal effects of ultrasound enhanced the cell death. We hypothesized that thermal and non-thermal effects are present with our LIPUS device in this experimental setup. This hypothesis is consistent with our observations in the *in vitro* setup using both the absorber and ultrasound (Fig. 5a & b). Cell viability assay results of the effect of DOX in the absence and presence of ultrasound are illustrated in Fig. 5. Fig. 5a presents the results for cells exposed to pure DOX and ultrasound, while Fig. 5b displays the results for cells treated with AuDOX nanoparticles and AuPVA nanoparticles [45] in which DOX was replaced by polyvinyl alcohol (PVA), a nontoxic, Food and Drug Administration (FDA)-approved polymer, under ultrasound exposure. We observed a significant difference in the cell viability results with the introduction of ultrasound and AuDOX. Pure DOX was highly cytotoxic, resulting in substantial cell death. In contrast, pure AuDOX exhibited lower toxicity because the DOX molecules were firmly adsorbed onto the gold surface. Cell death occurred over time due to the gradual passive release of DOX from the surface of the AuNPs. In both cases, ultrasound exposure following treatment with DOX or AuDOX led to significantly greater cell death compared to other groups, highlighting the synergistic effects of ultrasound and the therapeutic agents. In the Au-PVA + US group (third bar in Fig. 5b), a slight, non-significant decrease in cell viability was observed compared to the control and ultrasound-only groups. This suggests that, in the absence of drug loading, the physical interaction between AuNPs and ultrasound does not induce significant membrane disruption or cell damage.

**Fig. 6:**
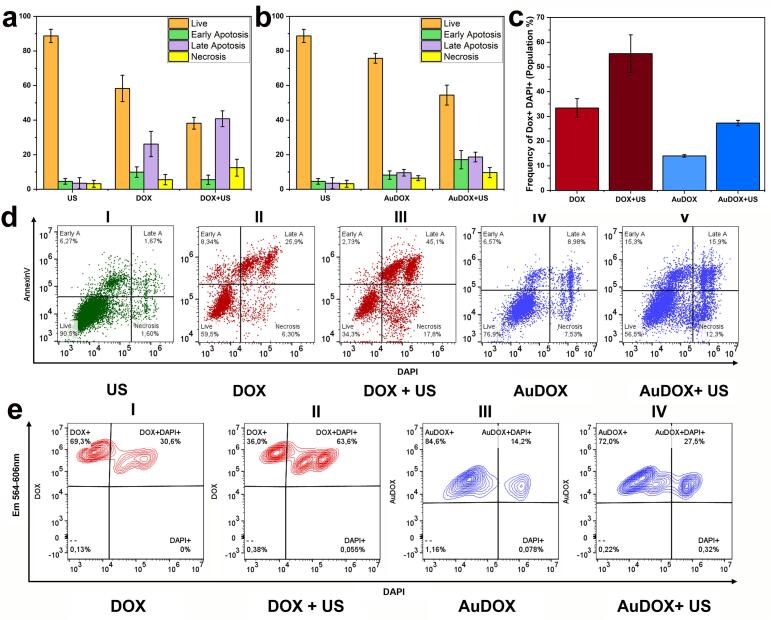
Flow cytometry-based Live-Dead analysis demonstrates increased cell apoptosis when simultaneously using (a) DOX and LIPUS exposure and (b) AuDOX and LIPUS exposure. (c) Percentage of DOX and DAPI double-positive cell population. Representative flow cytometry results from one of the three trials for the US, DOX, DOX + US, AuDOX, and AuDOX + US treatment conditions are shown in Fig. 6d (I)–(V), respectively. Live cells, which are Annexin V positive, are shown in the bottom left quadrant, while dead cells, which are DAPI positive, are displayed in the bottom right quadrant. The percentage of cells in each quadrant is indicated in the flow cytometry figures. Notably, the percentage of live cells in the control groups was 93.2 ± 5.1 %. These values were excluded from the figures to maintain consistency in the presentation of the experimental results. (e) Representative flow cytometry results from one of the three trials for the DOX, DOX + US, AuDOX, and AuDOX + US treatment conditions. The data show the percentage of DOX and DAPI double-positive cell populations and DOX + DAPI − cells. The error bar illustrates the mean ± standard deviation of three experimental measurements. The concentration of DOX is 9.5 µM in all relevant cases. Error bars represent the standard deviation of three experiments.

To further evaluate cell viability after treatment, two fluorescence dyes, Annexin V-FITC and DAPI, were employed. These dyes were chosen specifically to avoid interference and overlap with the broad emission and absorption spectra of DOX and AuDOX. Following treatment with DOX, AuDOX, and ultrasound, the cells were analyzed using flow cytometry. Fig. 6a, b, and d illustrate the distribution of live and dead populations under different treatments. The Annexin V-FITC positive live cells are in the bottom left quadrant, while dead cells, which are DAPI positive, appear in the bottom right quadrant. For the ultrasound, DOX, and DOX + ultrasound treatments, the percentages of live cells are 88.6 ± 3.8 %, 57.9 ± 7.6 %, and 38.8 ± 2.6 %, respectively (Fig. 6a). For the AuDOX, and AuDOX + ultrasound treatments, the percentages of live cells are 76.1 ± 3.1 %, and 54.6 ± 5.9 %, respectively (Fig. 6b). As a result, the outcomes of the DOX-only group and the AuDOX + ultrasound group are comparable, with the DOX + ultrasound group demonstrating the lowest viability. In addition, the percentages of cells in early and late apoptosis, as well as necrosis, are depicted in Fig. 6a and b. Fig. 6d (I)–(V) present detailed sample results from one of the three trials for each treatment condition, showing flow cytometry analysis for the following: US, DOX, DOX + US, AuDOX, and AuDOX + US.

We employed three primary treatment conditions: US, DOX, and AuDOX. Each treatment presented distinct interference with the flow cytometry channels and the dyes used due to fluorescence overlap between the drug DOX and the dyes, as well as nanoparticle scattering. We individually calibrated the gates for DAPI and Annexin-V staining under each treatment condition to account for this variability. This allowed us to accurately differentiate between the four cellular states: live cells, early apoptosis, late apoptosis, and necrosis. As a result, the gating boundaries in Fig. 6d(I)–(V) vary to reflect these specific adjustments. The data indicate that most monolayer cells remained viable and active after ultrasound exposure. However, the majority of cells entered the apoptosis phase after treatment with AuDOX + US. This suggests that a greater amount of DOX was internalized by the cells when loaded onto AuNPs and subjected to ultrasound (when quantifying DOX+/DAPI + population, Fig. 6c and Fig. 6e(III) & 6e(IV)). A similar trend was observed with DOX alone and DOX + US treatments (Fig. 6c and Fig. 6e(I) & e(II)). This result was expected, as the 48-hour incubation period post-treatment allowed sufficient time for the drug and nanoparticles to diffuse and penetrate the cells *via* passive uptake, enabling them to exert their cytotoxic effects.

**Fig. 7:**
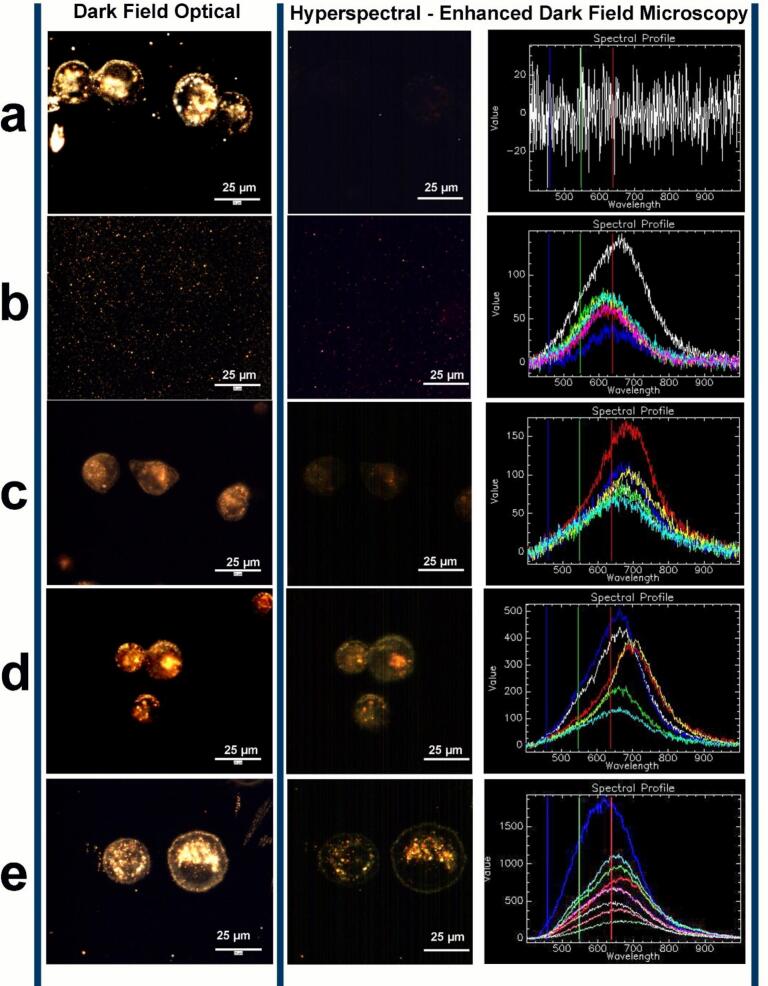
Representative dark-field optical and HSI micrographs of (a) MDA-MB-231 cells; (b) AuDOX nanoparticles in solution; AuDOX nanoparticles inside MDA-MB-231 cells (c) no ultrasound, (d) with ultrasound and no absorber, and (e) with ultrasound and absorber. Ultrasound activation initiated a substantial internalization of AuDOX nanoparticles in MDA-MB-231 cells, as evidenced by the formation of big clusters of AuDOX nanoparticles inside cells and thus increased the scattering intensity from those internalized nanoparticle clusters.

Hyperspectral imaging (HSI) and dark field microscopy provided non-destructive real-time imaging and spectral confirmation of AuDOX nanoparticles within live cells [46]. Although this was a qualitative analysis, it provided valuable insights into the enhanced uptake of AuNPs, as clusters of AuNPs were clearly visible inside living cells (Fig. 7c–e). Fig. 7a shows the dark field optical and hyperspectral images of untreated control cancer cells, where the cell membrane and other organelles scattered light, appearing as bright yellow-white against a dark background. The spectral intensity of scattered light from control cells, captured through HEDFM was very low compared to AuNPs, exhibiting a flat profile with considerable background noise. Fig. 7b shows the darkfield optical image of AuDOX nanoparticles (reflectance spectra) on a glass slide, where each bright orange/yellow dot represents mostly individual AuDOX nanoparticles or their small clusters. As most of the particles are either single or in small clusters, the intensity of scattered light was low, with a spectrum close to background noise. The HSI micrographs of ultrasound-mediated AuDOX uptake compared to AuDOX uptake in the absence of ultrasound are shown in Fig. 7d & e *vs*. Fig. 7c. The results indicate a significant increase in cellular uptake of AuDOX nanoparticles after applying ultrasound. The cell membrane appears bright white in dark field microscopy and typically exhibits a scattering spectrum with high noise levels (Fig. 7a), consistent with the literature [37]. In contrast, AuDOX nanoparticles display a sharp peak at around 500–700 nm (Fig. 7b & c) [47]. Following exposure to ultrasound, there is a significant increase in scattering intensity and a significant reduction in background noise levels (Fig. 7d & e), indicating the presence of a higher concentration of AuDOX nanoparticles and the formation of multiple AuDOX clusters of various sizes within the cellular environment [35]. Without the absorber, the primary mechanism is mechanical effects, as evidenced by the insignificant temperature increase shown in Fig. 4b, leading to enhanced cellular uptake. With the absorber, both mechanical and thermal effects from the ultrasound result in significantly higher cellular uptake than those without the absorber. This increase is attributed to ultrasound-mediated enhancement of endocytosis and sonoporation, thereby boosting cellular uptake through active and passive transport mechanisms. Given the size of MDA-MB-231 breast cancer cells (∼12–20 µm [48]) and the significantly smaller size of our nanoparticles (∼7–9 nm), coupled with the enhanced uptake under ultrasound exposure, a substantial number of AuDOX nanoparticles were able to penetrate the cell membrane.

## 4 Discussion

The primary objective of ultrasound-mediated NSDDSs is to use ultrasound that penetrates deep inside tumor tissue, enabling the precise and prompt release uptake of drugs from NSDDSs in a localized manner, thus increasing the cytotoxicity without escalating total drug dosage by enhancing the uptake of drugs and AuNPs. The synergistic combination of ultrasound with AuDOX nanoparticles or free DOX can potentially improve cancer therapy's efficacy. By overcoming biological barriers to cellular uptake, ultrasound enhances the intracellular delivery of therapeutic agents, either DOX or AuDOX nanoparticles.

In therapeutic ultrasound applications for mechanical stimulation and drug delivery, selecting the appropriate transducer frequency (1–3 MHz) is important, as it determines the depth of penetration and the biological effects induced. Lower frequencies (e.g., 0.5 MHz) are effective for applications requiring ultrasound to pass through high-impedance barriers like the skull, as they experience less attenuation and allow for deeper tissue penetration. However, the higher frequencies (1–3 MHz) absorb more energy in soft tissues, leading to stronger biological responses, making them more suitable for targeted therapeutic effects. Our choice of 1 MHz balances penetration and biological response, making it suited for treating a wide range of deep-seated organs and tissues. At this frequency, ultrasound waves penetrate sufficiently deep while delivering enough energy to induce cellular responses such as fibroblast activation and enhanced tissue repair. Compared to lower frequencies, 1 MHz provides more efficient mechanical stimulation and drug uptake in soft tissues, increasing its therapeutic impact. By selecting 1 MHz, we ensure that the ultrasound reaches deeper anatomical structures while promoting biologically effective energy absorption that enhances healing, increases blood flow, and facilitates drug delivery across a broader target area.

To identify suitable ultrasound parameters, we evaluated cellular responses across nine preset duty cycle combinations and power available on our LIPUS transducer [41]. Five power levels (1.8, 3.2, 3.8, 6.2, and 8.4 W) were selected based on their differential effects on cell viability. Notably, 1.8 and 3.2 W caused less than a 10 % reduction in MDA-MB-231 viability, indicating minimal cell damage. The 3.2 W setting—corresponding to high power and 50 % duty cycle—was chosen for further drug release and delivery analysis in this study due to its balanced combination of effective ultrasound exposure for drug release and cellular safety. Future studies will expand the parameter space to better understand how varying LIPUS conditions affect different cell types and therapeutic outcomes. As described above, unfocused ultrasound generated by the LIPUS transducer was employed to trigger drug release from AuNPs, capitalizing on both thermal effects (within the mild hyperthermia range) and mechanical effects (including ARF and acoustic streaming). The cell viability tests demonstrated a significant enhancement of cell killing after applying ultrasound and therapeutic agents (both DOX alone and AuDOX nanoparticles). This synergistic effect is primarily attributed to the mechanical and thermal effects of unfocused ultrasound at low intensities, ranging from 0.1 W/cm^2^ to 1.7 W/cm^2^, on the cells seeded on the membrane in the present study.

Pulsed ultrasound is often the preferred mode for therapeutic applications involving mechanical stimulation, drug delivery, and drug release, due to its ability to deliver biologically effective energy with minimal thermal effects and broad tissue coverage. Unlike continuous wave ultrasound, which can lead to excessive tissue heating, pulsed ultrasound delivers short bursts of acoustic energy that allow tissues to dissipate heat between pulses. This makes it suitable for stimulating cellular activity without damaging surrounding healthy tissue [49]. The unfocused nature of the ultrasound beam allows for energy distribution across a wider area, which is particularly beneficial when treating diffuse or irregularly shaped tissue regions. The unfocused beam can achieve therapeutic goals such as promoting healing in musculoskeletal injuries or enhancing drug transport in soft tissues. In contrast, focused ultrasound modes like low-intensity focused ultrasound (LIFU) are designed for precise targeting of small regions and are better suited to applications like neuromodulation or ablation, where spatial localization and precision are critical. Unfocused ultrasound transducers emit a wider beam of acoustic energy that diverges as it travels through the tissue, and it can cover a larger area with a more uniform pressure distribution compared to the sharp peak of focused ultrasound. In drug delivery and drug release applications, such as releasing DOX from AuNPs, pulsed ultrasound facilitates controlled mechanical effects and potentially mild local heating, both of which can trigger the release of therapeutic agents without destabilizing drug carriers or harming tissue.

In our *in vitro* setup (Fig. 2b), the temperature of degassed water was maintained at 37 °C to mimic the average human body temperature, suggesting that initial observations of the effects were solely due to the mechanical effects induced by ultrasound. However, temperature measurements (Fig. 4b) indicated that the presence of an ultrasound absorber on top of the medium resulted in a temperature rise to around 44.4 °C, which falls within the higher end of the hyperthermia range [50]. The hyperthermic condition is due to the setup design, which incorporates an ultrasound absorber positioned atop a small volume of cell culture medium in a well (5 mL). The absorber is placed directly in contact with the medium's surface, ensuring no air gap at the interface. This ultrasound absorber attenuates and converts ultrasound energy into heat, generating a hyperthermic environment. Thus, our setup permits the study of both the thermal and mechanical effects of ultrasound.

The present study demonstrates that ultrasound significantly enhances the cellular uptake of AuNPs and free drug through thermal and non-thermal mechanisms. These possible mechanisms might include bubble-based (cavitation) and non-bubble-based (ARF and acoustic streaming) methods for transient membrane permeabilization [27]. Cavitation, which involves the formation of gas-filled pockets, generates localized mechanical forces and micro-jets that can disrupt cell membranes, creating transient pores which facilitate nanoparticle entry, *i.e.*, sonoporation. For non-cavitation effects, ARF can generate shear stress at fluid/tissue interfaces *via* non-uniform ARF exposure on tissues, cell-to-cell junctions, and cellular interfaces [51]. LIPUS applies a non-uniform exposure to the monolayer cell culture (Fig. 3c), which leads to a gradient of shear stresses applied to cells. This shear stress can also produce temporary pores in cell membranes [25]. Sonoporation is a passive transport process where ultrasound induces partial cell membrane disruption, creating multiple pores ranging from tens of nanometers to several micrometers. The membrane disruption significantly enhances the permeability of the cell membrane to macromolecules [52]. If these pores are small, the living cell can repair them by a phenomenon known as “reparable sonoporation.” Consequently, sonoporation holds significant promise for shuttling large therapeutic molecules and nanoparticles for applications in drug delivery [52]. The widely accepted biophysical mechanism behind sonoporation involves shear stress caused by the rapid decline in microstreaming velocity near the cell boundary [53]. The sonoporation process bypasses the normal endocytic pathways typically involved in cellular uptake. The cell phospholipid bilayer membrane comprises a mix of lipids and proteins that form a highly selective permeability obstacle regulating the migration of various substances in and out of the cell.

Under normal conditions, endocytosis is the active transport mechanism by which AuDOX nanoparticles cross the cell membrane. However, in this study, the biophysical interactions of cell membranes are significantly influenced by ultrasound-induced effects like ARF, acoustic streaming and hyperthermia, enhancing AuNPs translocation across the membrane. For example, ARF can exert ultrasound-induced radiation forces that drive the movement of nanoparticles, improving their distribution within the intracellular environment [25,54,55]. The effect of ARF on AuNPs facilitates the manipulation of nanoparticle transport, contributing to improved interactions between nanoparticles and cell membranes as AuNPs enter the cells. In addition, ultrasound radiation pressure generates multidirectional acoustic microstreams that produce high shear stress, thereby enhancing the release and delivery of therapeutic agents [1]. Furthermore, acoustic streaming induced by ultrasound creates fluid motion that increases the contact frequency between nanoparticles and cell membranes, further promoting nanoparticle transport [28,56].

On the other hand, in an *in vitro* environment, hyperthermia can significantly enhance cellular uptake through several mechanisms, including increased cell membrane permeability and enhanced endocytosis. Elevated temperatures increase the fluidity of cell membranes, making them more permeable to therapeutic agents [57]. This enhanced permeability facilitates the entry of these agents into the cells. Increased temperatures can promote the expression of heat shock proteins, which protect cells from thermal stress and assist in the folding and trafficking of endocytosed nanoparticles [58]. Additionally, hyperthermia can lead to the reorganization of the cytoskeleton, facilitating vesicle formation and movement within the cell. This shows hyperthermia not only aids in the physical disruption of the cell membrane but also actively engages cellular machinery to optimize nanoparticle internalization, offering a potent strategy for enhancing the efficacy of nanoparticle-mediated drug delivery systems [59]. The combination of the increased fluidity of cell membranes due to hyperthermia and the mechanical effects of ultrasound could result in synergistic cellular uptake.

The observed 5 °C temperature increase, in conjunction with the ultrasound-induced mechanical effects generated in our *in vitro* setup with the absorber, likely contributed to the enhanced cellular uptake of AuDOX nanoparticles (Fig. 7e) compared to the setup without the absorber (Fig. 7d). . In both conditions, these effects promoted the formation of multiple clusters of AuDOX nanoparticles. In these experiments, a mechanism other than endocytosis, sonoporation, has also likely occurred. Previous studies on ultrasound-mediated intracellular delivery of liposomes and dextrans with microbubbles for high-intensity focused ultrasound have shown that complete inhibition of endocytosis does not result in the complete blockage of cellular uptake, suggesting that pore formation is part of the mechanism [25]. In this study involving low-intensity unfocused ultrasound with drug-loaded AuNPs, we demonstrated that the enhanced cellular uptake is likely driven by a combination of mechanisms: sonoporation facilitated by ARF and acoustic streaming, as well as enhanced endocytosis, which is presumed to be due to hyperthermia, with additional contributions from ARF and acoustic streaming. In summary, we have limited evidence to that supports the occurrence of bubble-mediated sonoporation in our studies. However, it is plausible that sonoporation mediated by ARF and acoustic streaming played a significant role. ARF and acoustic streaming could contribute in two ways: (a) by increasing shear stress that leads to the formation of pores in the cell membrane and (b) by inducing biochemical changes, such as gene upregulation, that enhance endocytosis.

The mechanisms underlying the effects of low-intensity ultrasound irradiation in cancer cell treatment are still being explored [49]. In our study, we focused on the roles of acoustic streaming and ARF rather than cavitation. Notably, our mechanical index is 0.3 below the cavitation threshold (MI = 0.5) [41,60]. Acoustic streaming can facilitate the transport of molecules, including drugs, to and from the cell membrane, enhancing the therapeutic effects. Fluid flow due to acoustic streaming can also generate mechanical stress on a cell membrane, inducing biological effects [61]. The ARF (on the order of a few Pa in this work) generates mechanical pressure on the cell membrane, which can lead to the activation of ion channels and changes in intracellular calcium levels, potentially enhancing drug uptake and promoting cell death pathways. Although the ultrasound beam was substantially larger than individual cells, the resulting ARF exceeds thresholds for eliciting mechanosensitive responses such as ion channel activation, cytoskeletal remodeling, and membrane deformation. Given the 10-minute exposure duration, these mechanical stimuli are expected to induce biologically relevant effects at the cellular level through strain gradients and indirect shear stresses. While the precise molecular mechanisms remain an area of ongoing research, our findings suggest that these mechanical forces are likely key contributors to the observed outcomes in cancer cell treatment.

**Fig. 8:**
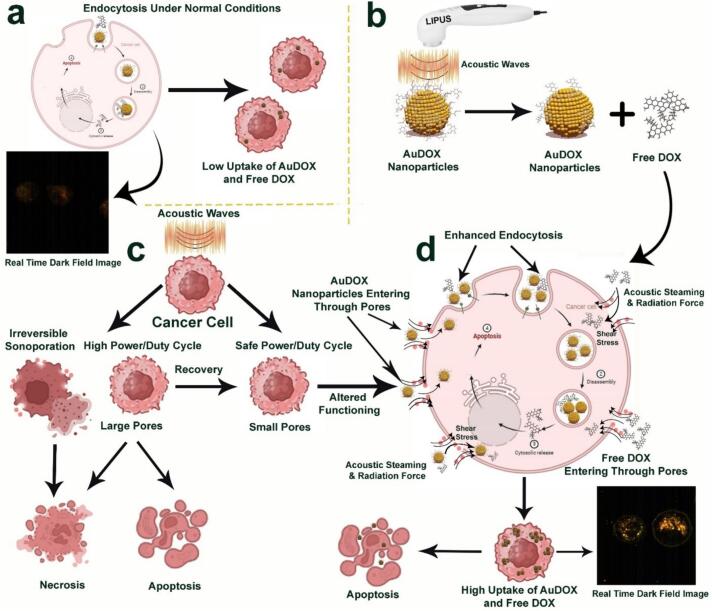
Schematic of mechanisms involved in drug release and enhanced cellular uptake of AuDOX in response to ultrasound. (a) Under normal conditions, the uptake of AuDOX nanoparticles and free DOX by cancerous cells occurs primarily through endocytosis, resulting in lower nanoparticle uptake. This is illustrated by the release of DOX molecules from AuNPs at normal body temperature, which is also evident in the dark field image showing low light scattering by AuNPs, indicating low uptake. (b) Ultrasound affects AuDOX nanoparticles by causing the release of DOX from the surface of the AuNPs, as explained in our recently published papers [5,21,31]. (c) The effect of high power and safe power ultrasound on cancerous cells. High-power ultrasound can create large or small pores or may cause irreversible sonoporation and cell trauma, leading to necrosis or apoptosis. In contrast, safe power ultrasound results in the formation of small pores through a phenomenon called sonoporation. (d) The formation of small pores alters the functioning of cancerous cells, leading to enhanced endocytosis and providing an alternative route for the entry of AuDOX nanoparticles and free DOX. This results in significant uptake of free DOX and AuDOX nanoparticles, clearly visible in the dark field image of the cancerous cell after ultrasound treatment. The notable temperature increase in our setup significantly boosted cellular uptake and AuNP clustering, indicating the role of both sonoporation and enhanced endocytosis. Enhanced uptake was driven by a combination of sonoporation facilitated by ARF and acoustic streaming and endocytosis primarily induced by hyperthermia, with additional contributions from ARF and acoustic streaming. This figure was created with BioRender.com.

As part of our findings, we propose a schematic representation (Fig. 8) illustrating the mechanisms involved in drug release and the enhanced cellular uptake of DOX and AuDOX in response to LIPUS. This schematic highlights the interplay between ultrasound-induced mechanical and thermal effects, along with cellular and nanoparticle behaviors, offering a visual understanding of how LIPUS enhances drug delivery and promotes more efficient cellular internalization. Under normal conditions, cancer cells primarily uptake AuDOX nanoparticles and free DOX through endocytosis, resulting in lower nanoparticle uptake, as shown by minimal light scattering in dark field images. Ultrasound enhances this process by promoting DOX release from AuNPs, as detailed in previous studies [5,21,31]. High-power ultrasound can induce large pores and cell damage, leading to necrosis or apoptosis, whereas safe-power ultrasound creates small pores through sonoporation, enhancing endocytosis. This combination of sonoporation and hyperthermia significantly increases the uptake of AuDOX nanoparticles and free DOX, as evidenced by increased cellular uptake and AuNP clustering observed in dark field images following ultrasound treatment. The enhanced uptake is driven by sonoporation and acoustic streaming, with hyperthermia further boosting endocytosis.

Flow cytometry was performed 48 h post-treatment to assess overall therapeutic efficacy and long-term cellular responses to the LIPUS-triggered nanoparticle drug delivery system. This time point enables comprehensive evaluation of cumulative cellular uptake and cytotoxicity. However, it is acknowledged that some cells may have undergone lysis or structural disintegration within this interval, potentially limiting their detection *via* flow cytometry. Although appropriate for observing downstream biological effects, this timing may not adequately reflect the early-phase mechanistic interactions associated with ultrasound-enhanced internalization. Future studies will incorporate earlier post-treatment time points to more accurately delineate the temporal dynamics of ultrasound-mediated uptake and intracellular trafficking.

The cellular response to ultrasound may differ significantly across cell types due to variations in membrane and mechanical properties. Cellular responses to ultrasound may vary markedly across cell types due to differences in membrane composition and thresholds for mechanobiological activation. These include stress thresholds to activate mechanosensitive ion channels, induce membrane deformation and cytoskeletal remodeling, or elicit shear-induced poration. While this study focused on the therapeutic effects of ultrasound on cancer cells, we acknowledge the importance of evaluating its efficacy and safety in other cell types, such as neurons and fibroblasts, to ensure biocompatibility and better tailor ultrasound-based strategies for diverse biological targets. This will be explored in detail in future investigations.

In conclusion, a combination of thermal and mechanical effects induced by low-intensity ultrasound exposure increases cell membrane permeability and fluidity. This phenomenon enhances the uptake of nanoparticles and free drug molecules into cells through actively and passively enhanced transport mechanisms facilitated by ultrasound exposure. In addition, ultrasound can induce the activation of cellular processes involved in endocytosis while changing the kinetics of the endocytosis process. Therefore, low-intensity unfocused ultrasound represents a promising adjunctive modality for enhancing the cellular uptake of drug-loaded AuNPs in cancer cells. Through its multifaceted mechanisms of action, ultrasound overcomes barriers to nanoparticle delivery and fosters the intracellular accumulation of therapeutic payloads. Given the accessible ultrasound exposure parameters required for mechanical effects and hyperthermia, this method can potentially be translated to *in vivo* and clinical settings. In such settings, where there will be no absorber to induce hyperthermia, the same transducer can be utilized interchangeably in pulsed and continuous modes: pulsed for generating mechanical effects and continuous for inducing thermal effects.

## Financial disclosure

This research was funded by Ontario Research Fund – Research Excellence (ORF-RE #RE02-032), the Natural Sciences and Engineering Research Council of Canada (NSERC) Alliance grant (ALLRP 556270-20) and NSERC Discovery grants that were awarded to J. Tavakkoli (RGPIN-2022-03799) and M.C. Kolios (RGPIN-2022-04143). F. Moradi Kashkooli is also supported by an NSERC Banting Postdoctoral Fellowship (funding reference no. 186566) administered by the Government of Canada. Additional funding support through a research contract from Toronto Poly Clinic Inc. is also acknowledged. The authors have no other relevant affiliations or financial involvement with any organization or entity with a financial interest in or financial conflict with the subject matter or materials discussed in the manuscript apart from those disclosed.

## CRediT authorship contribution statement

**Farshad Moradi Kashkooli:** Writing – review & editing, Writing – original draft, Visualization, Validation, Software, Project administration, Methodology, Investigation, Formal analysis, Data curation, Conceptualization. **Anshuman Jakhmola:** Writing – review & editing, Visualization, Methodology, Investigation, Formal analysis, Data curation, Conceptualization. **Graham A. Ferrier:** Writing – review & editing, Visualization, Software, Methodology, Investigation. **Monika Lodyga:** Writing – review & editing, Visualization, Software. **Kevin Rod:** Resources, Funding acquisition. **Jahangir (Jahan) Tavakkoli:** Supervision, Resources, Methodology, Funding acquisition, Conceptualization. **Michael C. Kolios:** Writing – review & editing, Supervision, Resources, Methodology, Investigation, Funding acquisition, Conceptualization.

## Declaration of competing interest

The authors declare the following financial interests/personal relationships which may be considered as potential competing interests: Hossein Zereshkian, Jahangir Tavakkoli, and Kevin Rod are involved with the patented LIPUS device used in the study (#CA3026302C, Current Assignee: Toronto Poly Clinic Inc., 2022). All other authors declare no competing interests.
